# A thesaurus for a neural population code

**DOI:** 10.7554/eLife.06134

**Published:** 2015-09-08

**Authors:** Elad Ganmor, Ronen Segev, Elad Schneidman

**Affiliations:** 1 Department of Neurobiology, Weizmann Institute of Science, Rehovot, Israel; 2 Department of Life Sciences, Zlotowski Center for Neuroscience, Ben-Gurion University of the Negev, Beer-Sheva, Israel; University of California, San Diego, United States

**Keywords:** neural code, information, noise, entropy, natural stimuli, metric, retina, salamander

## Abstract

Information is carried in the brain by the joint spiking patterns of large groups of noisy, unreliable neurons. This noise limits the capacity of the neural code and determines how information can be transmitted and read-out. To accurately decode, the brain must overcome this noise and identify which patterns are semantically similar. We use models of network encoding noise to learn a thesaurus for populations of neurons in the vertebrate retina responding to artificial and natural videos, measuring the similarity between population responses to visual stimuli based on the information they carry. This thesaurus reveals that the code is organized in clusters of synonymous activity patterns that are similar in meaning but may differ considerably in their structure. This organization is highly reminiscent of the design of engineered codes. We suggest that the brain may use this structure and show how it allows accurate decoding of novel stimuli from novel spiking patterns.

**DOI:** http://dx.doi.org/10.7554/eLife.06134.001

## eLife digest

Our ability to perceive the world is dependent on information from our senses being passed between different parts of the brain. The information is encoded as patterns of electrical pulses or ‘spikes’, which other brain regions must be able to decipher. Cracking this code would thus enable us to predict the patterns of nerve impulses that would occur in response to specific stimuli, and ‘decode’ which stimuli had produced particular patterns of impulses.

This task is challenging in part because of its scale—vast numbers of stimuli are encoded by huge numbers of neurons that can send their spikes in many different combinations. Furthermore, neurons are inherently noisy and their response to identical stimuli may vary considerably in the number of spikes and their timing. This means that the brain cannot simply link a single unchanging pattern of firing with each stimulus, because these firing patterns are often distorted by biophysical noise.

Ganmor et al. have now modeled the effects of noise in a network of neurons in the retina (found at the back of the eye), and, in doing so, have provided insights into how the brain solves this problem. This has brought us a step closer to cracking the neural code. First, 10 second video clips of natural scenes and artificial stimuli were played on a loop to a sample of retina taken from a salamander, and the responses of nearly 100 neurons in the sample were recorded for two hours. Dividing the 10 second clip into short segments provided a series of 500 stimuli, which the network had been exposed to more than 600 times.

Ganmor et al. analyzed the responses of groups of 20 cells to each stimulus and found that physically similar firing patterns were not particularly likely to encode the same stimulus. This can be likened to the way that words such as ‘light’ and ‘night’ have similar structures but different meanings. Instead, the model reveals that each stimulus was represented by a cluster of firing patterns that bore little physical resemblance to one another, but which nevertheless conveyed the same meaning. To continue on with the previous example, this is similar to way that ‘light’ and ‘illumination’ have the same meaning but different structures.

Ganmor et al. use these new data to map the organization of the ‘vocabulary’ of populations of cells the retina, and put together a kind of ‘thesaurus’ that enables new activity patterns of the retina to be decoded and could be used to crack the neural code. Furthermore, the organization of ‘synonyms’ is strikingly similar to codes that are favored in many forms of telecommunication. In these man-made codes, codewords that represent different items are chosen to be so distinct from each other that even if they were corrupted by noise, they could be correctly deciphered. Correspondingly, in the retina, patterns that carry the same meaning occupy a distinct area, and new patterns can be interpreted based on their proximity to these clusters.

**DOI:** http://dx.doi.org/10.7554/eLife.06134.002

## Introduction

Noise is prevalent in the nervous system, from ion channels through synapses and single neurons, and up to the system level (Mainen and Sejnowski, 1995; Schneidman et al., 1998; Rieke et al., 1999; Osborne et al., 2005; Faisal et al., 2008; Ala-Laurila et al., 2011). Consequently, even when presented with identical stimuli, the brain may receive different spiking patterns from the sensory neurons (Mainen and Sejnowski, 1995; Berry et al., 1997; de Ruyter van Steveninck et al., 1997; Reich et al., 2001). The nature of neural noise thus determines how information is encoded in the brain (Borst and Theunissen, 1999; Stocker and Simoncelli, 2006; Cafaro and Rieke, 2010; Rolls and Treves, 2011), and how it might be read out from neural activity (Warland et al., 1997; Dan et al., 1998; Vargas-Irwin et al., 2010). To correctly decode, the brain must overcome this noise (Osborne et al., 2005; Sreenivasan and Fiete, 2011).

In engineering, codes are designed to solve this problem by choosing codewords that are far apart in the space of patterns, relative to the typical noise levels. That way, if noise corrupts a message, it would still be easily distinguishable from the noisy variants of other codewords (Cover and Thomas, 1991; Sreenivasan and Fiete, 2011; Curto et al., 2013). It is not clear however, how this issue is resolved in the brain, or how it affects the design of the neural code, where information is carried by the joint activity patterns of large groups of noisy neurons (Nicolelis et al., 1995; Maynard et al., 1999; Mazor and Laurent, 2005; Fujisawa et al., 2008; Pillow et al., 2008; Truccolo et al., 2010; Ganmor et al., 2011a; Harvey et al., 2012). It is clear that the nature of correlations among neurons is central in shaping the code's capacity and content, in particular in the context of noise (Zohary et al., 1994; Zemel et al., 1998; Abbott and Dayan, 1999; Diesmann et al., 1999; Sompolinsky et al., 2001; Schneidman et al., 2006; Pillow et al., 2008; Shlens et al., 2009; Ganmor et al., 2011b; Schwartz et al., 2012; Granot-Atedgi et al., 2013). However, the functional role of these correlations in encoding information by large populations has been heavily debated (Nirenberg et al., 2001; Amari et al., 2003; Pola et al., 2003; Schneidman et al., 2003a, 2006; Averbeck et al., 2006; Tkacik et al., 2006; de la Rocha et al., 2007; Pillow et al., 2008; Ecker et al., 2010; Ohiorhenuan et al., 2010; Oizumi et al., 2010; Ganmor et al., 2011a), partly because of the difficulty to study them directly at the network level and the limitations of generalizing from small groups of cells to large ones.

To uncover the structure and content of neural population codebooks, we must be able to quantify the similarity between population activity patterns. In other words, we need to understand network noise well enough to know which patterns are likely to be interchanged in encoding the same stimulus. Several studies suggested intuitive and computationally efficient measures of spike train similarity (Victor and Purpura, 1997; van Rossum, 2001; Houghton and Sen, 2008). However, they mostly focused on single neurons and often assumed the functional form of similarity between responses. Despite their computational simplicity, it is not clear whether such simple metrics can adequately represent similarity between neural responses. The Hamming distance, for example, is a very intuitive measure of similarity between population activity patterns, simply defined as the number of neurons that switch state from spiking to silence and vice versa. Yet, it measures similarity in form, or syntactic similarity, but not necessarily similarity in meaning, that is, semantic similarity (Huth et al., 2012). This would be analogous to suggesting that ‘night’ and ‘light’ are similar in meaning because they differ by just one letter. Extending such metrics to large populations requires additional assumptions on the way the similarity measures between different cells should be combined.

We suggest here that the semantic similarity between neural activity patterns can be measured by comparing what each pattern tells the brain about the stimulus. We demonstrate that in the vertebrate retina, responding to natural and artificial stimuli, we can estimate this similarity between any pair of population responses, using models of population encoding noise. Using this similarity structure, or ‘thesaurus’, we can map the semantic organization and content of the population codebook and show how it enables the accurate decoding of novel population patterns.

## Results

**Figure 1.**
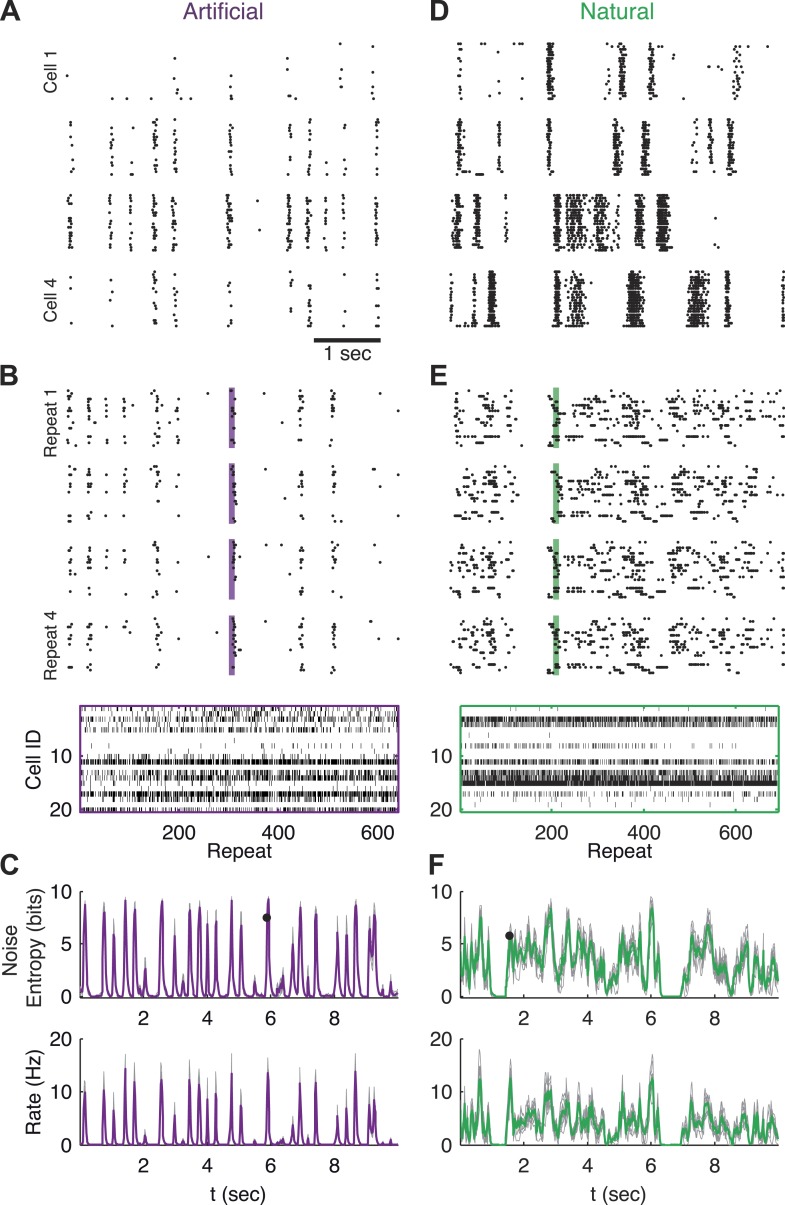
Population responses to natural and artificial stimuli are noisy. (**A**) Subset of the responses of four retinal ganglion cells to 20 repeats of the same artificial video clip (out of 641 altogether). Each block corresponds to one cell, each line to a single repeat; black dots mark spike times. (**B**) Top: Response of a population of 20 retinal ganglion cells to four repeats of the same stimulus as in **A**. Here, each block represents the response of the entire population in a single trial and each line represents a single cell. Bottom: All 641 population responses of the 20 cells (across repeats) for one time point marked by the shaded bar in the raster plot above. Black ticks represent spikes; each vertical slice in the plot is the population response on a single trial and is therefore a single sample from the conditional distribution of responses given the stimulus presented at that point in time *P*(*r*|*s*). (**C**) Top: Conditional entropy of the population response patterns, given the stimulus, as a function of time for a 10 s artificial video clip repeated hundreds of times. Thin gray lines correspond to different groups of 20 cells; Average over 10 groups is shown in purple. Bottom: The average firing rate, for the same stimulus, shown as a function of time. (**D**–**F**) Same as **A**–**C** but for a natural video clip (with 693 repeats). **DOI:** http://dx.doi.org/10.7554/eLife.06134.003

To study the variability, or noise, which is inherent to encoding of sensory stimuli by large populations of neurons, we presented isolated salamander retinas with over 600 repeats of the same 10 s long video clips (one natural video and one artificial spatially uniform and temporally Gaussian flicker video), and recorded the joint response of dozens of retinal ganglion cells (see ‘Materials and methods’). Each video was presented continuously in a loop for ∼2 hr. This allowed us to collect hundreds of neural population responses evoked by the exact same visual stimulus (including identical stimulus history up to several minutes). Figure 1A shows examples of the stochastic, or noisy, responses of single neurons from the population to the same stimulus segment (see also Mainen and Sejnowski, 1995; Berry et al., 1997).

In order to examine the responses at the population level, we defined the population activity pattern *r(t)* as the binary vector of the instantaneous states of each neuron: the *i*th entry in the vector, r^i^, being 1 if the *i*th neuron spiked, and 0 if it was silent (responses were binned in 20 ms bins; for other bin sizes see Figure 5—figure supplement 4). Note that each time point, *t,* corresponds to a particular segment of the video (*t* runs from 0 to 10 s—the length of the video), and thus to a particular visual stimulus which we denote as *s(t)*. For a 10 s long video, binned at 20 ms, we have 500 such stimuli.

By collecting the responses recorded at the same time point in the video across many repeated presentations, we obtain samples from the conditional distribution of activity patterns *r* given a stimulus *s*, denoted *P*(*r*|*s*). This distribution is commonly referred to as the ‘encoding distribution’ of the stimulus. Figure 1B shows samples of the joint responses of 20 neurons (the population size we focus on in this study) across the repetitions of the video, reflecting the wide variety of different population activity patterns evoked by the same stimulus.

We quantified the variability of the population patterns that encoded the same stimulus by the entropy, *H*, of the encoding distribution, *P*(*r*|*s*), which measures the ‘width’ or richness of the distribution (see ‘Materials and methods’). Figure 1C shows the entropy of *P*(*r*|*s*) as a function of time (or, equivalently, of stimulus), along with the instantaneous population rate, reflecting that the variability of the population responses is high when the population's firing rate goes up.

### Measuring semantic similarity between population activity patterns

The encoding noise determines which population patterns may be used to encode the same stimulus and thus implicitly defines similarity between patterns. We submit that two neural activity patterns are similar, in terms of their meaning, to the extent that they convey similar information to the brain. This constitutes *semantic* similarity between population patterns, which does not necessarily rely on any *syntactic* similarity (for example total spike count). Intuitively, this is analogous to synonyms in natural language, which may not look similar, but still have the same meaning (Pereira et al., 1993).

The meaning of a neural activity pattern *r* is given by what it tells the brain about its corresponding input, or in the case of sensory systems, the potential stimuli that could have resulted in that response. Since the mapping between stimulus and response is probabilistic, the meaning of *r* is given by the probability distribution over stimuli conditioned on that response, namely *P*(*s*|*r*). We therefore defined the distance between two neural population activity patterns *r*_k _and *r*_l _as the dissimilarity between *P* (s|r_k_) and *P *(s|r_l_),

(1) $$d\left(r_{\text{k}},r_{\text{l}}\right)={\text{D}}_{\text{JS}}\left(P\left(s|r_{\text{k}}\right)||P\left(s|r_{\text{l}}\right)\right),$$

where D_JS_ is the Jensen-Shannon divergence between the distributions (see ‘Materials and methods’)—a symmetric measure of dissimilarity that varies between 0, for identical distributions, and 1, for non-overlapping distributions. Here, 0 would imply that the two activity patterns are perfect ‘synonyms’ and have identical meaning, whereas 1 would imply completely different meaning.

Rather than directly estimating *P*(*s*|*r*), which is challenging both experimentally and statistically, we can instead estimate *P*(*r*|*s*) and infer *P*(*s*|*r*) through Bayes' rule, namely *P*(*s*|*r*) = *P*(*r*|*s*)*P*(*s*)/*P*(*r*), where *P*(*s*) is the distribution over stimuli (set to be a uniform distribution over all video frames used in the experiment), and *P*(*r*) is the distribution of the neural responses (calculated by marginalizing over stimuli, $P\left(r\right)=\underset{s}{\sum }P\left(s\right)P\left(r|s\right)$). Our experimental design, in which we presented many repeats of the same video, allowed us to draw hundreds of samples from *P*(*r*|*s*), for every *s*. However, due to the exponential number of possible population activity patterns one cannot directly sample this distribution in full even for groups of 20 neurons. Instead, we construct accurate models of population encoding noise, which we then use to estimate *d*(*r*_i_,*r*_j_) for any pair of population patterns.

### Population responses to ‘interesting’ stimuli exhibit strong network noise correlations

The properties of encoding noise, and in particular, the magnitude and importance of correlations between neurons in encoding a stimulus, commonly known as ‘noise correlations’, have been heavily debated (Nirenberg et al., 2001; Schneidman et al., 2003a; Ohiorhenuan et al., 2010; Oizumi et al., 2010). For pairs of neurons, average noise correlations are typically weak (Cafaro and Rieke, 2010; Ecker et al., 2010), implying that pairs of neurons are not far from being conditionally independent given the stimulus. If groups of cells were encoding information independently, that is, if $P\left(r|s\right)=\Pi_{i}P\left(r^{\text{i}}|s\right)$ for large populations, then *P*(*r*|*s*) would be defined by the individual and independent noise of each neuron and would be easily learned from the noise of each neuron *P*(*r*^i^|*s*). However, weak pairwise correlations do not imply that large groups are conditionally independent in encoding stimuli (Schneidman et al., 2006; Pillow et al., 2008; Vidne et al., 2012; Granot-Atedgi et al., 2013). Moreover, noise correlations are often estimated on average, over a range of different stimuli, and it is not clear what low average noise correlations imply for population encoding of specific stimuli.

**Figure 2.**
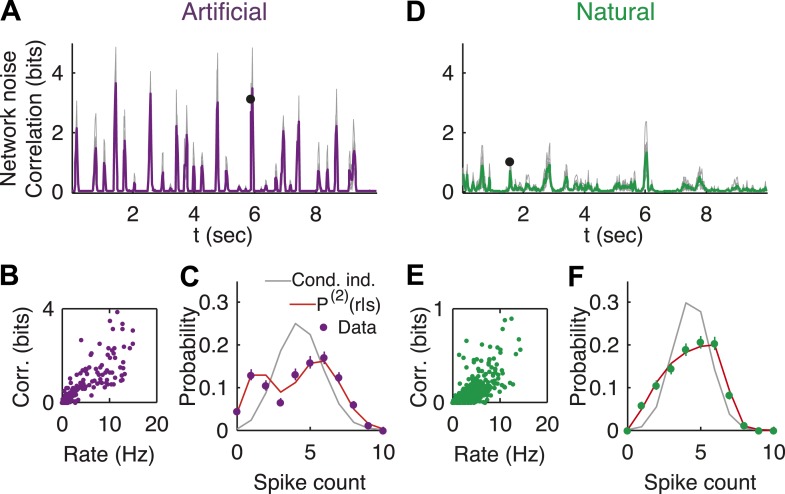
Strong noise correlations, at the population level, at interesting times in the video. (**A**) Population noise correlation, measured by the multi-information over the conditional population responses, at each point in time in response to an artificial video. Thin gray lines correspond to individual groups of 20 cells; average over groups is shown in purple. (**B**) Population noise correlation as a function of average population firing rate for one representative group. Interesting events in the video evoke a vigorous response by the retina, characterized by strong network correlations. (**C**) Distribution of spike counts across different repeats of the same stimulus for the time point marked by black dot in **A**. Purple dots correspond to empirical estimates, gray line is what we would expect if neurons were conditionally independent, given the stimulus; and red line is the prediction of the maximum entropy pairwise model. (**D**–**F**) Same as **A**–**C** but for a natural video clip. **DOI:** http://dx.doi.org/10.7554/eLife.06134.004

We therefore estimated the noise correlations at the population level, for each stimulus *s*. Specifically, we quantified the correlation of the population in encoding the stimulus *s*, by the multi-information, $I\left(r|s\right)=H\left[P^{ind}\;\left(r|s\right)\right]-H\left[P\left(r|s\right)\right]$ (Amari, 2001; Schneidman et al., 2003b), where $H\left[P^{ind}\;\left(r|s\right)\right]$ is the entropy assuming neurons are independent given the stimulus—$P^{\text{ind}}\left(r|s\right)=\Pi_{i}P\left(r^{\text{i}}|s\right)$, and H[P(r|s)] is the entropy of the joint population response to the stimulus *s* (estimated following Strong et al., 1998). We note that *I*(*r|s*) measures the total correlations of all orders among the cells. We found that most stimuli did not evoke a substantial response from the retina, which gave rise to low noise correlation in the network for these stimuli. However, when something ‘interesting’ happened in the video and the ganglion cells increased their firing rates, we found a sharp increase in the degree of network noise correlations for both natural and artificial videos (Figure 2A,B; see Figure 2—figure supplement 1 for analysis of sampling properties). Thus, while on average the network noise correlations may be weak, the population was strongly correlated and far from conditionally independent in response to interesting stimuli. In other words, for these stimuli, the variability or noise at the level of the network is significantly reduced compared to what would be expected from the apparent noise level of individual cells.

**Figure 2—figure supplement 1.**
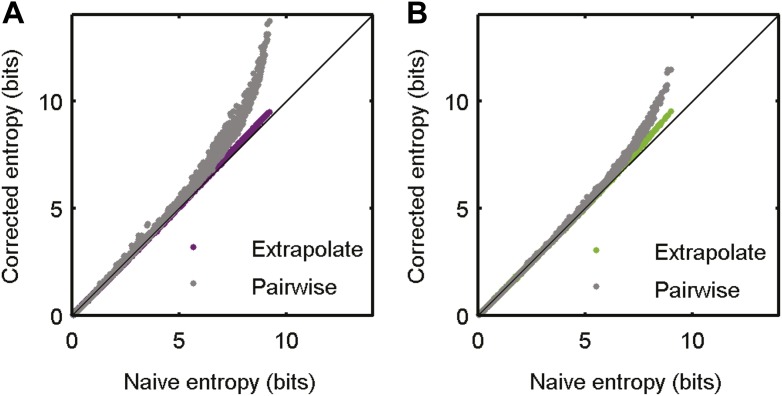
Accurate sampling of entropy. (**A**) Pairwise maximum entropy upper bound on entropy (gray) and the extrapolation corrected noise entropy (Treves and Panzeri, 1995; Strong et al., 1998) (purple) are shown as a function of the naive noise entropy estimate. Each dot corresponds to the distribution at a single time point in the artificial video; black line marks identity. The bias corrections are on the order of a few percent at most. (**B**) Same as **A**, but for data taken from the natural video data set (693 repeats). **DOI:** http://dx.doi.org/10.7554/eLife.06134.005

**Figure 2—figure supplement 2.**
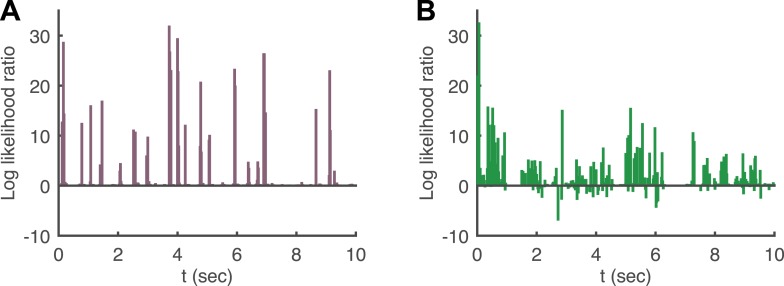
Log likelihood ratio of the pairwise model and the conditionally independent model. (**A**) A pairwise (second order maximum entropy as in the text) and an independent model (product of marginals) was fit to each time point (equivalently stimulus) in the artificial video using 90% of video repeats. The log likelihood ratio of the two models (log[Likelihood_Pairwise_/Likelihood_Cond. − Indep._]) is plotted as a function of time (equivalently stimulus), for the held out 10% of repeats. The likelihoods are similar much of the time, corresponding to low firing epochs, but for many points in time the likelihood of the pairwise model can be orders of magnitude higher. (**B**) Same as **A**, for the natural video. **DOI:** http://dx.doi.org/10.7554/eLife.06134.006

Indeed, conditionally independent models of population encoding (which assume no noise correlations) fail exactly at such interesting stimuli (Pillow et al., 2008; Granot-Atedgi et al., 2013), reflecting that in order to accurately characterize the population responses and the noise, we need a model that takes into account correlations between the cells that depend on the stimulus. We extended the framework of maximum entropy models for neural activity patterns (Schneidman et al., 2006; Shlens et al., 2009), to the population responses to each stimulus (Granot-Atedgi et al., 2013): for each stimulus, s, we fit the minimal model that has the same stimulus dependent firing rates and pairwise noise correlations observed over the repeats of that stimulus. This stimulus-dependent pairwise maximum entropy model is given by

(2) $$P^{\left(2\right)}\left(r\text{|}s\right)=\frac{1}{Z\left(s\right)}\text{exp}\left(\underset{i}{\sum }\alpha_{i}\left(s\right)r^{i}+\underset{i<j}{\sum }\beta_{ij}\left(s\right)r^{i}r^{j}\right),$$

where *α*_*i*_(*s*) and *β*_*ij*_(*s*) were fit to obey the constraints. We found that *P*^(2)^(*r*|*s*) gave relatively accurate models of the distribution of patterns that the population displayed across repeats, for the different stimuli (Figure 2—figure supplement 2), in agreement with Granot-Atedgi et al. (2013). In particular, they captured accurately the strong network noise correlations (Figure 2C).

### A thesaurus for a neural population code

We then use the distributions *P*^(2)^(*r*|*s*) that describe the population encoding noise, to estimate the dissimilarity, *d*(*r*_i_,*r*_j_), between any pair of patterns over the population in a reliable and precise manner. Since there are over a million possible population activity patterns for 20 neurons, we present here only the similarity matrix of all the population patterns that were observed in the test data (half of the data, which was randomly selected and not used to learn the similarity between activity patterns).

**Figure 3.**
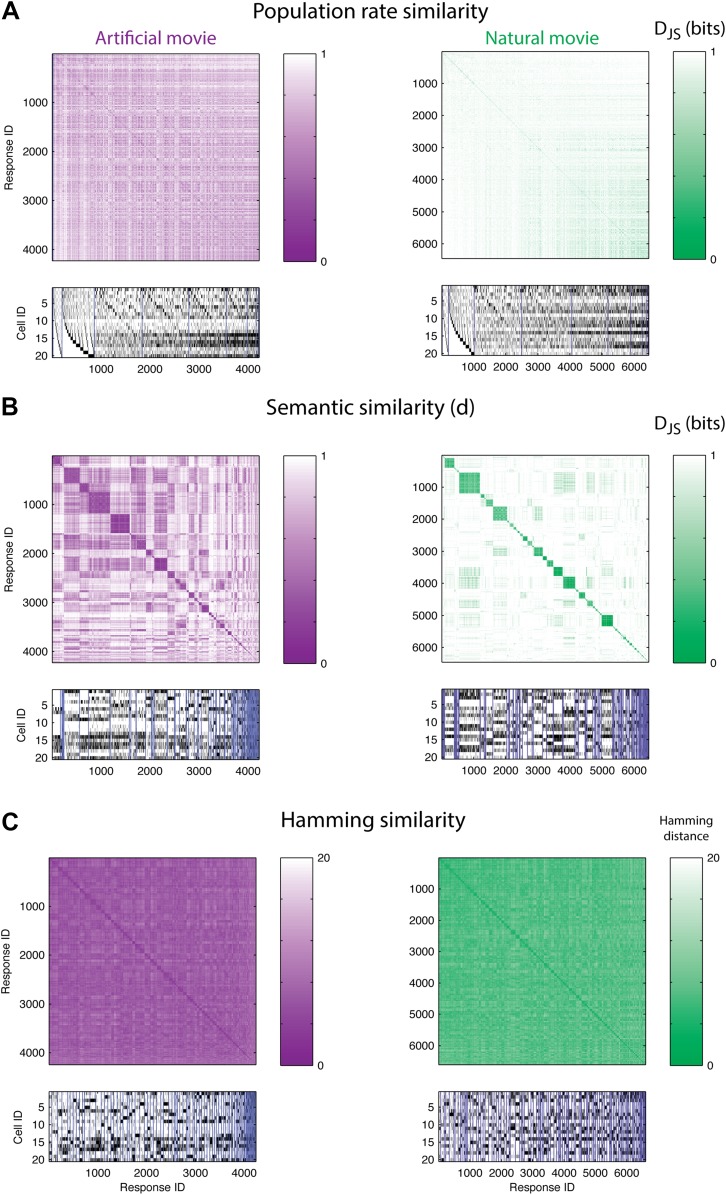
The population code of the retina is comprised of clusters of responses with highly similar meaning. (**A**) Top: Similarity matrices of the population responses of representative groups of 20 neurons to an artificial (left) and natural (right) video. Each entry in the matrix corresponds to the similarity, *d* (see text), between two population responses observed in the test data (responses shown at bottom). Matrix rows (and columns) are ordered by total spike count in the population responses. Bottom: The population responses corresponding to the entries in the matrix; black ticks represent spikes. Each column is a population activity pattern corresponding to the matrix column directly above. Blue lines mark borders between different clusters. The lack of structure in the matrices implies that population responses with similar spike counts do not carry similar meanings. (**B**) Same as **A**, only here the matrix is clustered into 120 clusters. Matrix rows (and columns) are ordered such that responses from the same cluster appear together. A clustered organization of the population code is clearly evident. (**C**) Same as **B**, but using the Hamming distance between population responses, instead of the similarity measure *d*. A simple measure of syntactic similarity does not reveal the underlying clustered organization of the code. **DOI:** http://dx.doi.org/10.7554/eLife.06134.007

If stimuli were represented by overall population rates (Bohte et al., 2000; Amari et al., 2003; Loebel et al., 2007; Schwartz et al., 2012), then responses containing the same number of spiking neurons would be similar, and thus would have a small *d* between them. Figure 3A shows the similarity matrix *d*(*r*_i_,*r*_j_) for one representative group of 20 cells, where matrix rows (and columns) stand for individual population patterns and are ordered according to the total number of spikes in each response. The lack of structure in the matrices for both artificial and natural stimuli shows that similar spike counts do not imply similar meaning.

**Figure 3—figure supplement 1.**
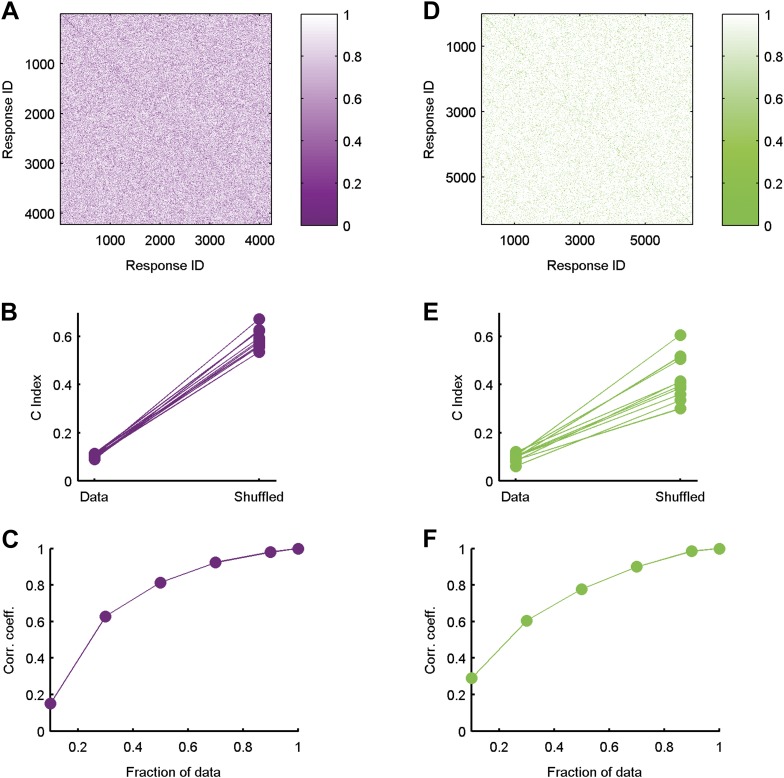
Clustered organization is highly significant. (**A**) The similarity matrix used in Figure 3 in the main text was first randomly shuffled and only then clustered. Clearly the grouping structure has disappeared, suggesting the structure is a property of the code's organization and not the values in the matrix or the power of the clustering algorithm. (**B**) For each of the ten 20 neuron groups we calculated the C Index (Hubert and Schultz, 2011) (left, labeled ‘Data’), which measures goodness of clustering; a smaller C Index corresponds to better clustering. For each matrix, we then measured the C Index for 100 randomly shuffled versions and took the minimal value of all shuffled matrices (right, labeled ‘Shuffled’). None of the shuffled values came close to the real values, indicating a p-value smaller than 0.01 for each matrix. (**C**) Correlation coefficient between similarity matrices estimated using different fractions of data. Overall we had 641 repeats of the artificial video. Using 70% of the repeats, we are at ∼0.9 correlation with our estimates using all available repeats (Data are for the same matrix shown in Figure 3 of main text). (**D**–**F**) Same as **A**–**C** but for data taken from the natural video data set. **DOI:** http://dx.doi.org/10.7554/eLife.06134.008

**Figure 3—figure supplement 2.**
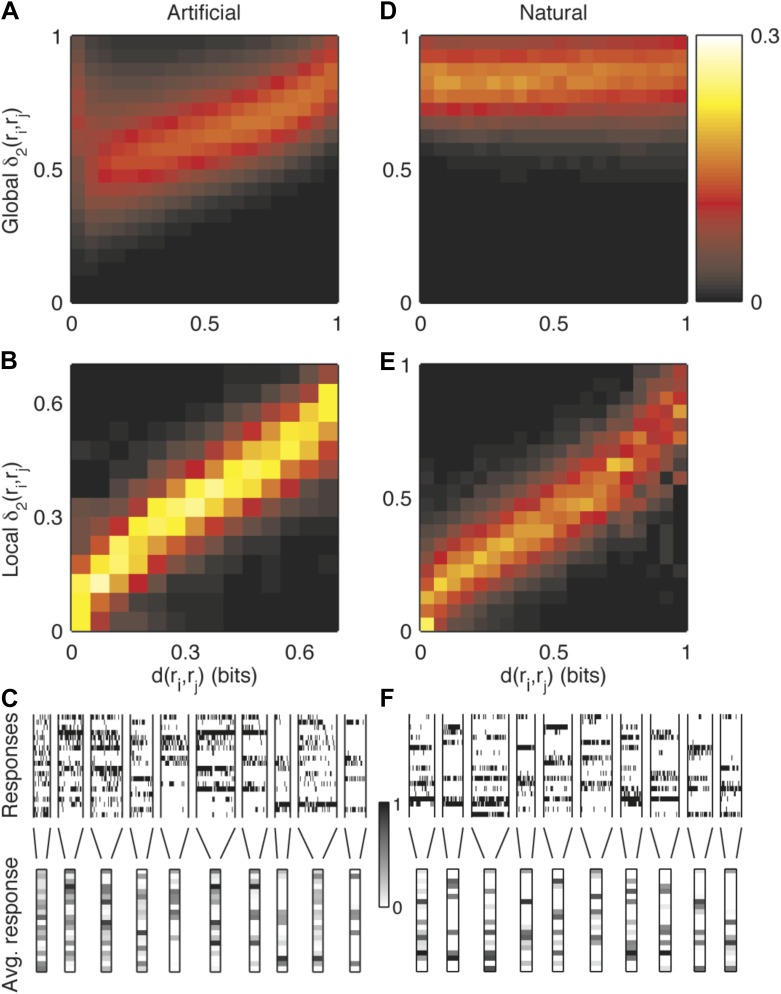
Semantic similarity between population patterns can be explained by a simple local similarity measure, but not by a global similarity measure. (**A**) We sought a simple formula to approximate the similarity measure *d*, in the spirit of the measures described in Victor and Purpura (1997); van Rossum (2001); Houghton and Sen (2008). The Hamming distance gave a very poor approximation of similarity and even a measure which gave a different weight to each neuron, $\delta_{1}\left(r_{i},r_{j}\right)=\underset{\mu }{\sum }w^{\mu }{\left(r_{i}^{\mu }-r_{j}^{\mu }\right)}^{2}$, performed very poorly. Shown is a joint histogram of the similarity values *d*(*r*_i_,*r*_j_) (x-axis) and the corresponding values predicted by a global second order similarity model $\delta_{2}\left(r_{i},r_{j}\right)=\underset{\mu }{\sum }\underset{\nu }{\sum }w^{\mu \nu }{\left(r_{i}^{\mu }-r_{j}^{\nu }\right)}^{2}$ (y-axis. *w* parameters fit to train data; results for cross-validated test data are shown) for the responses of a representative group of 20 neurons (same as in Figure 3) to an artificial video. For clarity, values were normalized about the y-axis, such that each vertical slice sums to one. (**B**) Joint histogram of the similarity values of pairs of responses from the same cluster (x-axis) and the similarity predicted by a local second order model for similarity, that is, *δ*_2_ applied independently to each cluster. Other details as in **A**. These results suggest that noise is highly stimulus dependent and cannot be accurately described by a global, stimulus independent, model. Yet, for a given cluster, we can accurately describe its similarity neighborhood, using the appropriate set of single neurons and neuron pairs. (**C**) In support of the previous conclusion, we found that close inspection of large response clusters reveals an obvious structure within clusters. Many of the clusters of similar responses can be characterized as having very precise neurons (almost always spiking or almost always silent), alongside more noisy neurons, which appear to be nearly random within a cluster. These precise and noisy neurons differ from one cluster to another. Shown is a detailed view of the responses in a subset of the clusters, which contain between 20 and 50 different patterns and appear most frequently in the data. Top: All population responses belonging to each cluster (clusters separated by vertical lines). Each horizontal line corresponds to one neuron; each vertical slice is the response of the population to a single repeat. Bottom: The average response of each cluster. The spiking probability of each neuron is represented by its gray scale intensity (color bar: dark—high spike probability, light—low spike probability). (**D-F**) same as (**A-C**), but for a natural video stimulus. **DOI:** http://dx.doi.org/10.7554/eLife.06134.009

In clear contrast, when we used a hierarchical clustering algorithm on *d*(*r*_i_,*r*_j_) matrices and grouped responses together according to their similarity (see ‘Materials and methods’), we found that the ordered matrices exhibit an almost perfect block diagonal structure (Figure 3B; see Figure 3—figure supplement 1 for statistical analysis). Thus, the population code that is used by the retina both for artificial videos, and for natural videos, is arranged in sets, or clusters, of highly similar activity patterns, which are very different from all other patterns. We term these groups of highly similar responses neural ‘synonyms’, and by analogy we refer to the similarity measure *d* as a *neural thesaurus*.

We examined whether the *Hamming* distance, which is a simple and intuitive measure of similarity between population responses (the number of neurons that differ in their spiking or silence state), was sufficient to reveal the structure of the population codebook. We thus constructed the matrices of Hamming distances between all pairs of population activity patterns, and clustered the responses, using the same hierarchical agglomerative clustering. Figure 3C shows the Hamming matrix for the same group of 20 cells from a–d, where matrix rows (and columns) are ordered according to the clustering results. We did not find evident structure in the codebook used for natural stimuli, and only slightly more structure was apparent for the artificial stimuli. We emphasize that the results of Figure 3 were typical for many independent choices of groups of cells (as is summarized later in Figure 5).

These results reflect the importance of measuring similarity in meaning and not similarity in structure. Using a syntactic (structure based) measure, we would not have been able to uncover the clustered organization of the neural population code that the semantic similarity reveals. Patterns that belong to the same cluster do exhibit some shared structure, namely some of the neurons almost always fire in a specific cluster and others are always silent (Figure 3B, bottom). However, importantly, we found that the semantic similarity structure over all the patterns we observed could not be captured by a simple linear or bilinear function of the population patterns (Figure 3—figure supplement 2).

### Coding by clusters

The clustered structure of the neural population codebook suggests that the same stimulus may be represented by different, yet semantically similar population patterns, or synonyms. Such structure is commonly used in engineered codes in computer science and communications (Cover and Thomas, 1991; Sreenivasan and Fiete, 2011; Curto et al., 2013). This gives rise to two important predictions, which we confirmed by cross-validation, using novel (held out) test stimuli: 1. Responses to repeated presentations of a stimulus should come from the same cluster. 2. Responses from the same cluster should be nearly interchangeable.

**Figure 4.**
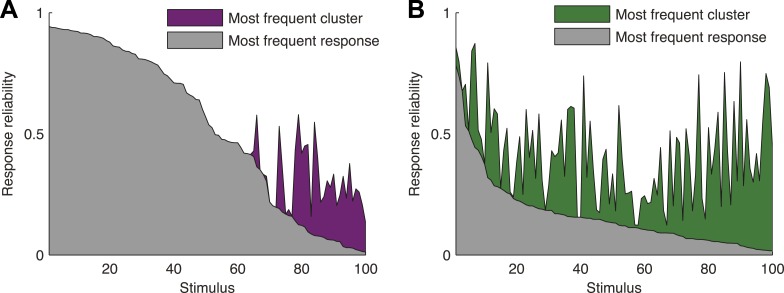
Responses to the same stimulus tend to come from the same cluster. (**A**) The probability of the most frequent response across video repeats is plotted as a function of stimulus identity in gray (stimuli are sorted by reliability). In purple, we plot the reliability of the clustered response, that is, the probability of observing a response from the most frequent cluster for each stimulus (clustering matrix presented in Figure 3). Only the 100 stimuli that evoked the strongest response are shown. Clearly, responses to the same stimulus tend to come from the same cluster, even when the most frequent single response occurs less than 20% of the time, thus the cluster code is far less noisy. (**B**) Same as **A** but for the natural video data set. **DOI:** http://dx.doi.org/10.7554/eLife.06134.015

To directly show the advantages of cluster-based encoding of information by the retina, we quantified the reliability of population patterns used to encode the same stimulus. Because of the noise, the responses to repeated presentations of the same stimulus are so variable that even the most frequent population pattern would occur only a handful of times (Figure 4A,B). However, the reliability of the population code is revealed when instead of focusing on individual patterns, we count how many of the population patterns evoked by the same stimulus belong to the same cluster. Notably, even when the population response was highly unreliable (i.e., the most frequent response pattern appeared less than 20% of the time), often over 70% of the observed responses would fall within a single cluster (mostly for the natural video), for the cross-validated test data (see Figure 4—figure supplement 1 for statistical analysis).

**Figure 4—figure supplement 1.**
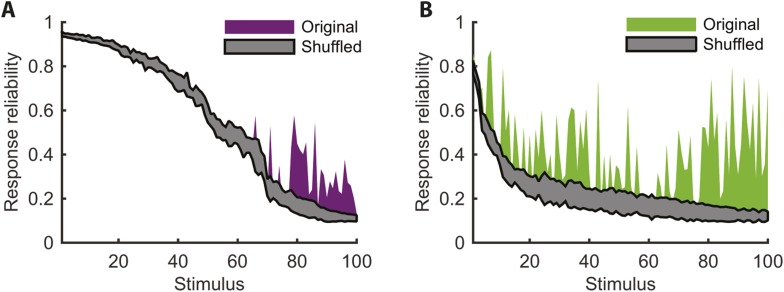
Increased reliability of clustered responses is highly significant. (**A**) Responses recorded at the same time point across video repeats cluster together significantly. Shown is the probability of the most frequent cluster, plotted as a function of stimulus id (purple; stimuli sorted by reliability), for the clustered response; that is, the probability of the most frequent cluster evoked by each stimulus (same as Figure 4A). For comparison, we also shuffled the cluster assignment of each response and repeated the analysis. Shown in gray is mean ± STD of the most frequent cluster in 100 randomly shuffled cluster assignments. Clearly, for many time points the high reliability of the clustered response is not merely a result of response grouping, but the tendency of responses from the same cluster to be associated with the same stimulus. (**B**) Same as **A**, but for natural video data set. **DOI:** http://dx.doi.org/10.7554/eLife.06134.016

### Clusters convey most of the information available about the stimulus

**Figure 5.**
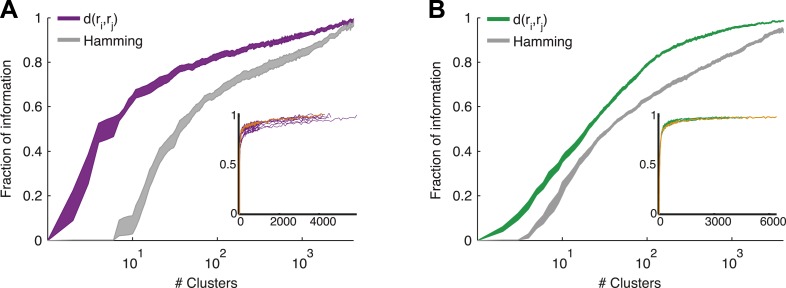
Cluster identity conveys most of the information about the stimulus. (**A**) The fraction of information retained about the stimulus when population responses are replaced with the label of the cluster they belong to, plotted as a function of the number of clusters used. Lines correspond to the average of 10 groups of 20 neurons, line widths represent SEM. Purple—clustering based on the similarity measure described in the text, gray—clustering based on the Hamming distance. Inset: Fraction of information as a function of the number of clusters on a linear scale. Individual groups are shown in gray, and the orange line marks the curve corresponding to the representative matrix from Figure 3. Very few clusters are required to account for most of the information, suggesting responses from the same cluster have a similar meaning. (**B**) Same as **A**, but for a natural video clip. **DOI:** http://dx.doi.org/10.7554/eLife.06134.010

The most direct test of ‘coding by clusters’ is to ask how much information about the stimulus would be lost if instead of knowing the precise population activity pattern (exactly which neurons spiked and which ones were silent) we only knew which cluster the pattern belongs to. We therefore compared the information that the full set of population responses *r* carry about the stimulus *I*(*s*;*r*), to the information that is carried just by knowing which cluster (out of *k* possible ones) the response belongs to, *I*(*s*;*C*_*k*_(*r*)). To that end, we labeled every population pattern in the test data according to its cluster identity, *C*_k_(r), based on the similarity structure learned from training data (see ‘Materials and methods’). To avoid any arbitrary assumptions about the number of clusters, we assessed how much information is carried by *k* clusters, for different values of *k* (Figure 5) on novel test data that was not used for learning the similarity structure. We found that ∼100 clusters were enough to account for over 80% of the information available about the stimulus in the detailed population patterns, for both types of stimuli. Importantly, clustering the responses based on the Hamming distance between them gave significantly worse results. This clearly reflects that responses in the same cluster have a similar meaning and can indeed be viewed as noisy variants of a noise-free codeword, similar to the ground states or attractors in a Hopfield model (Hopfield, 1982; Tkacik et al., 2006).

**Figure 5—figure supplement 1.**
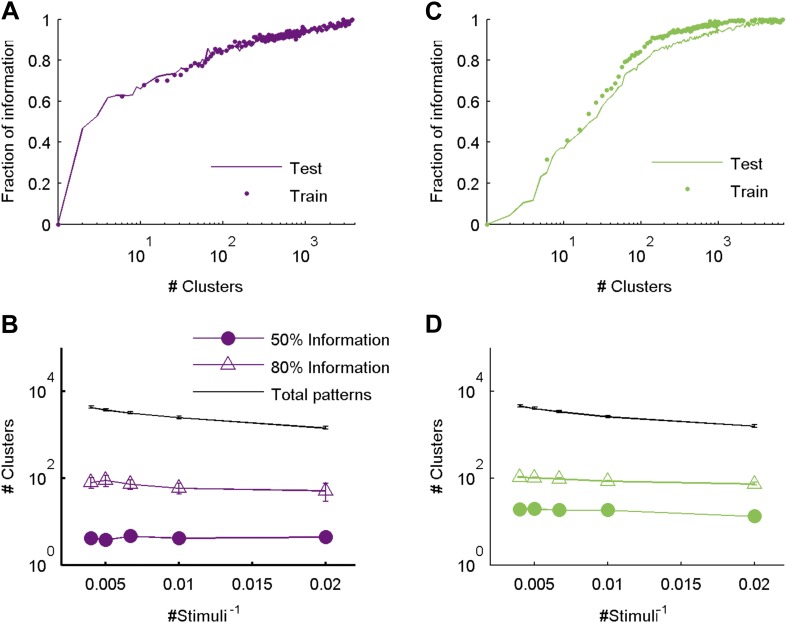
The similarity measure generalizes well across stimuli. (**A**) Fraction of information retained about the stimulus plotted against the number of response clusters. The similarity measure learned from the artificial video train data is applied either to cross-validated test data (same as Figure 5 of main text; purple line), or to the train data itself (purple dots). We see nearly identical performance on cross-validated and non cross-validated data. Shown is the same representative group of 20 neurons as in Figure 3. (**B**) The number of clusters required to recover over 50% (filled purple circles) or 80% (open triangles) of the information available about the stimulus, plotted as a function of the inverse number of stimuli in the test data (train data remained fixed), on a semi-logarithmic scale. Also shown, for comparison, is the overall number of observed patterns (black line). Depicted are average and SEM (may be smaller than markers) over 10 different groups of 20 neurons. As the number of stimuli in the test set increased, we saw a very mild increase in the number of clusters required to account for either 50% or 80% of the information. In fact, there was no significant increase in the number of clusters when the number of stimuli increased from 200 to 250 (p > 0.4, sign rank test), while the number of observed patterns grew significantly by over 500 (p < 0.01 sign rank test). This suggests that the clusters (or ‘code-words’) we identified are relevant to a wide range of stimuli within the same stimulus class. (**C**, **D**) Same as panels **A** and **B**, but for data taken from the natural video data set. **DOI:** http://dx.doi.org/10.7554/eLife.06134.011

**Figure 5—figure supplement 2.**
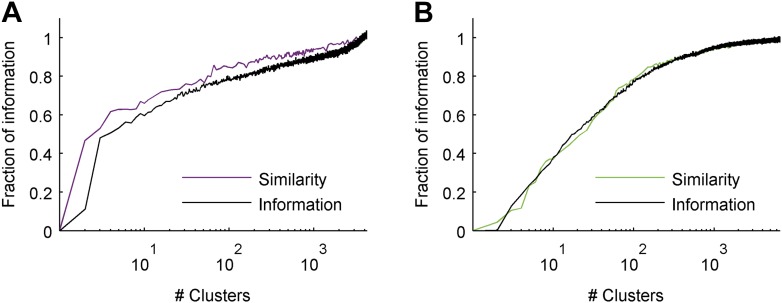
Clustering aimed at maximizing the mutual information yields similar results to clustering based on similarity alone. (**A**) Fraction of information retained about the stimulus plotted against the number of response clusters (full field flicker stimulus). Responses were either clustered using simple agglomerative clustering (purple. Same as in main text, see ‘Materials and methods’), or using agglomerative information bottleneck clustering (Slonim and Tishby, 1999), which explicitly aims to cluster responses such that maximal information about the stimulus is retained (black). Although, we would expect clustering aimed at information maximization to do a better job, after cross-validation (applying the clustering to novel responses to novel stimuli), we see that the simple similarity based clustering performs just as well. The data shown are for the same representative group as used throughout the main text. We note that results are shown for cross-validated test data, and that information bottleneck clustering is a greedy approach with no guarantee of optimality, thus it is possible for similarity based clustering to outperform information bottleneck clustering. (**B**) Same as **A**, but for the responses to a natural video stimulus. **DOI:** http://dx.doi.org/10.7554/eLife.06134.012

**Figure 5—figure supplement 3.**
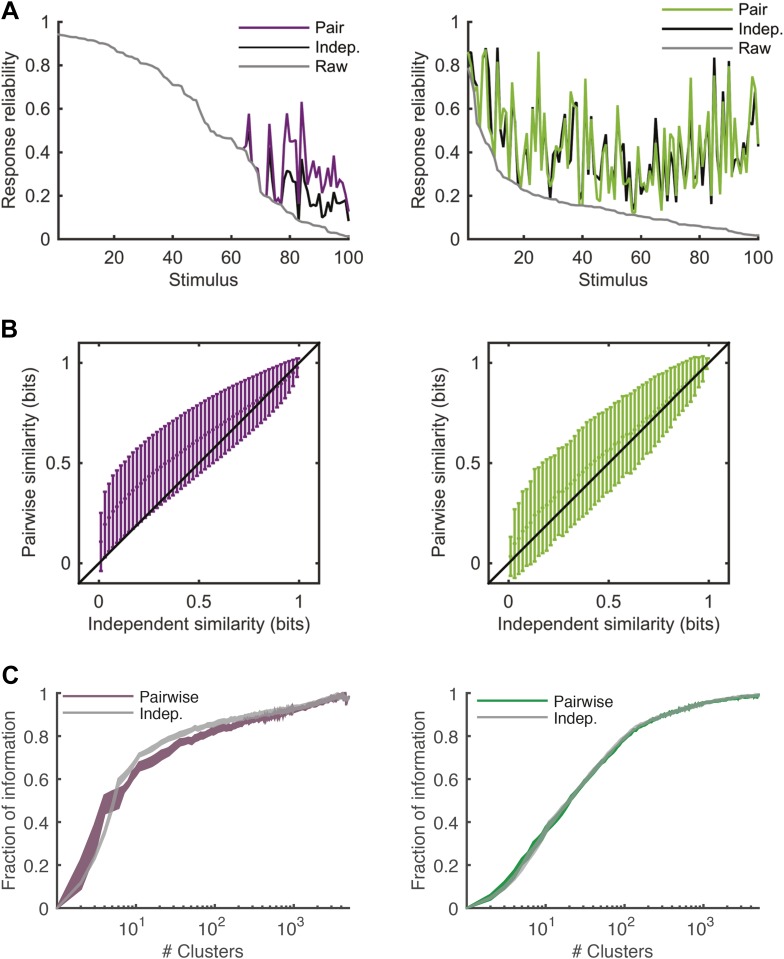
Comparing response similarity derived from the conditionally independent model and pairwise model. (**A**) Left: The probability of observing the most frequent response across repeats of the artificial video is plotted as a function of stimulus i.d. in gray; for clarity, the stimuli are sorted by reliability of their responses. In black is the reliability of the clustered response, that is, the probability of the most frequent cluster evoked by each stimulus, where responses were clustered by similarity derived from a conditionally independent model. In purple is the clustered reliability as derived from the pairwise model used in the main text (same as Figure 4). Only stimuli that evoked a strong response (at least one spike in over 75% of the repeats) are shown. Here, reliability is much higher when using the more accurate pairwise model to derive similarity between population responses. Right: Same as left, but for the Natural video data set. Here, differences between conditionally independent and pairwise models are less pronounced. (**B**) Left: Similarity derived from the conditionally independent model is directly compared with the similarity derived from the pairwise model, for test responses to the artificial video. Similarities between response pairs calculated using the conditionally independent model were binned (x-axis) and the mean and standard deviation of the similarity for the same pairs was calculated using the pairwise model (y-axis). Black line marks identity. Right: Same as left, but for the natural video. We see that similarity measured using the conditionally independent model is closer to that measured using the more accurate pairwise model under natural stimulation. (**C**) Left: same as Figure 5A, but here we compare the fraction of information as a function of number of clusters curve obtained by using the pairwise model (purple) and the one obtained using the conditionally independent model, in which noise correlations are ignored (gray). **DOI:** http://dx.doi.org/10.7554/eLife.06134.013

**Figure 5—figure supplement 4.**
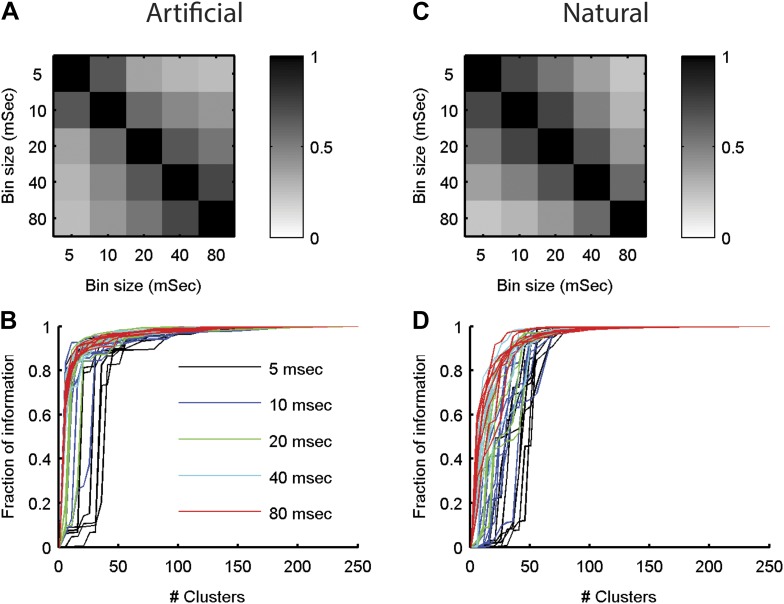
The neural ‘thesaurus’ remains stable across different bin sizes. (**A**) Correlation matrix between the similarity values estimated for all possible response pairs. For a given group of 8 neurons from the artificial video data set, we calculated the full similarity matrix over all 256 possible responses. This was done for bin sizes between 5 and 80 ms. We then calculated the correlation between the similarity matrices for each combination of bin sizes. Shown is an average across 8 randomly chosen groups of 8 neurons. The results indicate that except for extreme differences in bin size, we recover highly consistent similarity structures. (**B**) The fraction of information is shown as a function of the number of clusters in the similarity matrix (compare to Figure 5 in main text). Different bin sizes are indicated by colors. Different lines correspond to different individual 8 neuron groups (same groups as **A**). We see very similar results except for the very short time bin of 5 ms. (**C**, **D**) Same as **A** and **B** but for data taken from the natural video data set. **DOI:** http://dx.doi.org/10.7554/eLife.06134.014

We therefore conclude that grouping responses by their similarity in the training data identified clusters that conserved the information available about the stimuli in the test data, thus indicating that the similarity we measured is a general feature of the code and not limited to a particular set of stimuli. Importantly, all stimuli in the test data and many of the neural responses they evoked were not observed in the training data that we used to learn *d*(*r*_i_,*r*_j_). Thus, the limited number of stimuli and responses observed in the training data were sufficient to identify semantic clusters and predict the similarity between over a million possible neural responses evoked by a multitude of different possible stimuli, which were verified using the test data. We further point out that *d*(*r*_i_,*r*_j_) was so stable across time and for different selections of train and test data, that the information curves derived from clustering the cross-validated test data and the training data itself were nearly identical (Figure 5—figure supplement 1). In addition, the number of clusters required to convey 80% of the information seems to saturate with the size of the test set (Figure 5—figure supplement 1). We conclude that the similarity between neural responses does not rely on the specific stimuli we used and would generalize to other stimuli at least within the same stimulus class.

We further emphasize that clustering of the responses was based on similarity alone and was not optimized to maximize information in any way (which could therefore give even better results). Yet, even if we clustered patterns by explicitly trying to maximize the information, we achieved very similar results (Figure 5—figure supplement 2). This suggests that grouping responses simply by their semantic similarity may be nearly optimal from an information transmission standpoint.

We also estimated the semantic similarity between population patterns with simpler models for the population responses to the stimuli—using conditionally independent models of encoding, where for each stimulus *s* the response is described by $P\left(r|s\right)=\Pi_{i}P\left(r^{\text{i}}|s\right)$. Although these models give a different and less accurate estimate for the probability distribution than the pairwise model *P*^(2)^(*r*|*s*) (Figure 2), we find that grouping the population activity patterns using this model results in a similar clustered structure (see Figure 5—figure supplement 3). This may suggest that the codebook organization into clusters is sufficiently robust, so that even when using a less accurate model of the neural responses one can identify the organization of the response space (see more in the ‘Discussion’).

### Mapping the structure of the neural population codebook and its meaning

**Figure 6.**
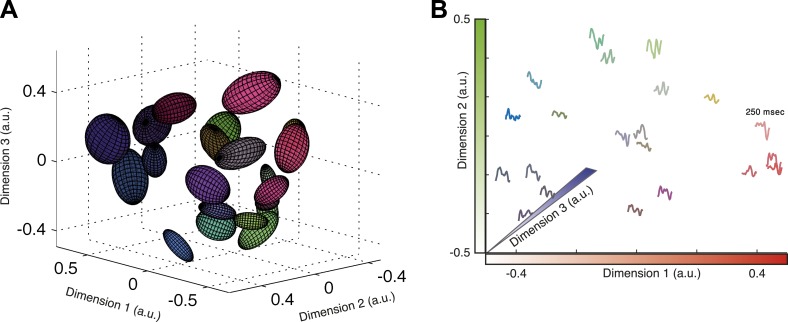
Response similarity predicts stimulus similarity. (**A**) The responses belonging to clusters that contain 30–300 patterns were embedded using *Isomap*. Each ellipse represents the 1 STD Gaussian fit to all responses belonging to a single cluster. The Euclidean distance in the plot approximates the similarity measure *d* (see text). The coordinates also correspond to the RGB value of each ellipse, thus nearby clusters share similar colors. Same representative group as in Figure 3. (**B**) Embedding of cluster triggered average waveforms in 2D Euclidean space. For each pair of clusters from panel **A**, we calculated the inter-cluster distance as the average similarity between pairs of responses, one from each cluster. Clusters were then embedded in 2D space using *Isomap* in a manner that approximates the calculated distances. Each cluster is represented by the mean stimulus that preceded (250 ms) responses belonging to that cluster. Thus, nearby waveforms belong to similar clusters. Clusters are colored as in panel **A**, therefore the blue channel corresponds to the third dimension of embedding not shown in the plot. **DOI:** http://dx.doi.org/10.7554/eLife.06134.017

Given that almost all information about the stimulus is carried by the identity of the cluster that a given activity pattern belongs to, we asked whether we can map the functional organization of the population codebook. We used *Isomap* (Tenenbaum et al., 2000) to present a low dimensional embedding of all the population responses associated with clusters that contain 30–300 responses in three-dimensional Euclidean space (Figure 6A; see ‘Materials and methods’ and Video 1 for the raw data points in 3D). *Isomap* is an embedding algorithm for high dimensional data that preserves the geodesic distance between points. While this embedding is imperfect (one would need more dimensions to achieve a nearly perfect embedding; see Figure 6—figure supplement 1), it provides an approximate view of the organization of the population code of a sensory system and coarsely reflects the ‘cloud’ of patterns that make up each cluster.

**Figure 6—figure supplement 1.**
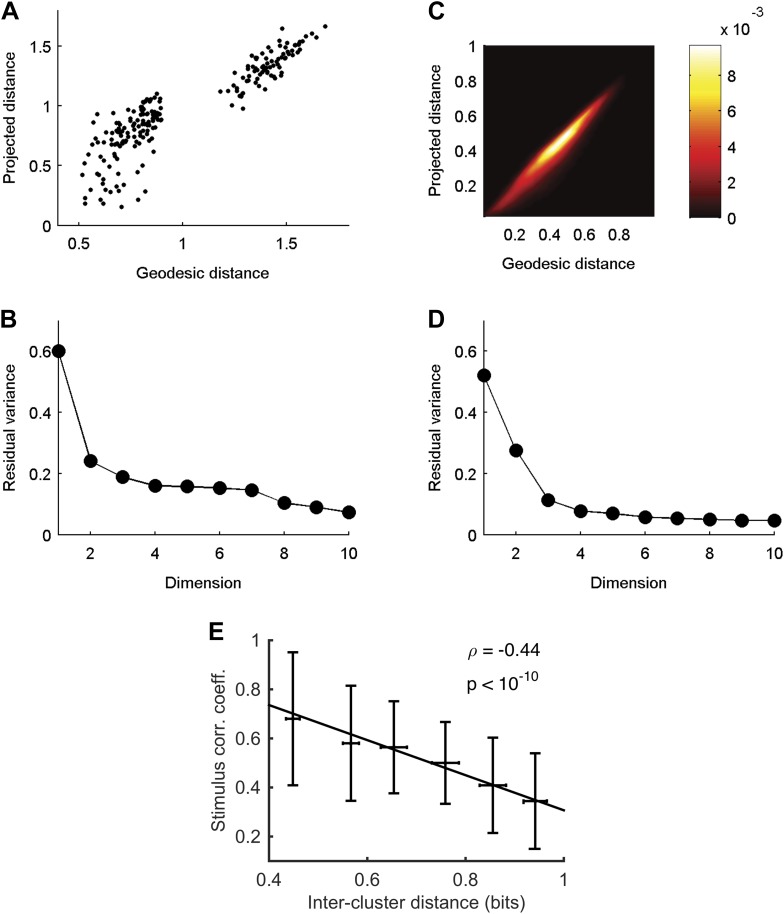
Cluster similarity implies stimulus similarity. (**A**) Geodesic vs embedded distances for the embedding shown in Figure 6A. The x-axis is the Geodesic distance between clusters (i.e., distances in the neighborhood graph; methods), vs the Euclidean distances after embedding in 3D. (**B**) Residual variance as a function of dimensionality for embedding of waveforms in Figure 6A. The residual variance is defined as 1 − *r*^2^(*d*_G_,*d*_Iso_), where *r* is the correlation coefficient, *d*_G_ is the geodesic distances between points as defined by the weighted neighborhood graph (Tenenbaum et al., 2000), and *d*_Iso_ is the Euclidean distances between points after embedding. (**C**) Same as **A**, but for embedding of responses in Figure 6B. Due to the large number of population responses embedded (2391), we show the joint histogram of the geodesic (x-axis) and embedded (y-axis) distance values. Colorbar represents frequency of occurrence of distance pairs. (**D**) Same as **B**, but for embedding of responses in Figure 6B. (**E**) For each pair of clusters shown in Figure 6A, we calculated the correlation coefficient between the cluster triggered average stimulus (the average of all stimuli preceding responses in a cluster) of each cluster, and plot it as a function of the distance between the clusters. Inter cluster distance was defined as the average similarity between pairs of responses, one from each cluster. Even though the correlation coefficient is not the ideal measure to quantify similarity among stimuli, we still see a clear and significant relationship between cluster similarity and stimulus similarity. Namely, clusters that are more similar (smaller values on the x-axis) have a higher correlation between their associated average stimuli. **DOI:** http://dx.doi.org/10.7554/eLife.06134.018

**Video 1.** Embedding of responses in 3D using *Isomap*. Each dot represents a single population response to the artificial video; the Euclidean distance between points approximates the similarity d between them. Similar to Figure 6A, only we explicitly plot every population activity pattern in each cluster. Colors represent different clusters and correspond to the colors in Figure 6A,B. **DOI:** http://dx.doi.org/10.7554/eLife.06134.019

To show the functional correlates of these clusters in coding, we present the similarity between clusters in terms of the stimuli that they encode. To that end, we quantified the distance between each pair of clusters, *C*_m_ and *C*_l_, by the average distance between all inter-cluster response pairs, ${\langle d\left(r_{i},r_{j}\right)\rangle }_{i\in C_{1},j\in C_{2}}$. We then embed the clusters in a Euclidean space according to the similarity between them (again using *Isomap*). Thus, in Figure 6B each cluster is represented by the average of its associated stimulus ensemble (we emphasize that the stimuli were taken exclusively from the test data). Hence, waveforms that are closer in space belong to clusters that are more similar. We find that response similarity measured from the training data predicts stimulus similarity in the test data (Figure 6—figure supplement 1 shows the correlation between cluster similarity and stimulus similarity; for more accurate metrics on stimulus space, see [Tkačik et al., 2013]). Thus, the similarity measure proposed here for the population responses captures similarity in meaning and generalizes across stimuli. We note that this analysis could only be carried out on the simple one dimensional full field video, as there is no evident way to reduce the dimensionality of natural stimuli.

### Decoding novel population patterns using the neural thesaurus

**Figure 7.**
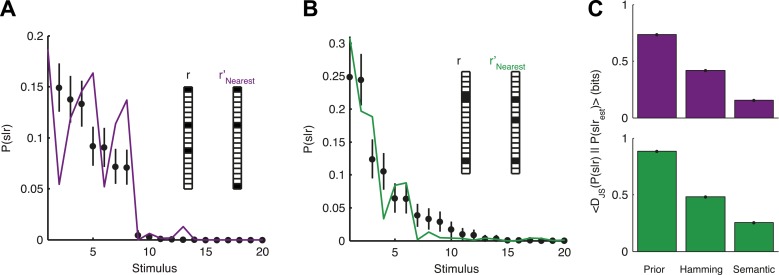
Accurate decoding of new stimuli from previously unseen population responses, using a neural thesaurus. (**A**) The conditional distribution over stimuli for one population response, *P*(*s*|*r*), to the artificial video is shown (black dots). *P*(*s*|*r*) can be well approximated by the conditional distribution over stimuli *P*(*s*|*r*′) where *r*′ is the response most similar to *r* according to the thesaurus *d* (‘*r*′_Nearest_’, purple line). Actual responses are shown as inset. The same representative group of 20 neurons shown in Figure 3 was used here. Error bars represent standard errors of the probability estimates **B**. Same as in **A**, but for a natural video clip. (**C**) Top: The average Jensen-Shannon divergence between the ‘true’ *P*(*s*|*r*) and the estimate described in panel **A** (Semantic), or for an estimate derived using the Hamming distance instead of our similarity measure (Hamming), for the artificial video data. Also shown is the average divergence from the prior over stimuli (Prior). Plotted are mean and standard errors (barely discernable) across all patterns that had at least one close neighbor (<0.25 bits away). Bottom: Same as above, but for the natural video data. Having a thesaurus markedly improves our ability to gain some knowledge about never before seen responses, compared to a naive prior, or even to using Hamming distance as a similarity measure. **DOI:** http://dx.doi.org/10.7554/eLife.06134.020

The organization of the retina codebook may also explain how the brain can decode novel stimuli from novel neural activity patterns. Namely, if we observe a response *r* that we have never seen before, we can now ask what similar responses tell us about the stimulus. Importantly, this can be done since *P*^(2)^(*r*|*s*) allows us to estimate *d*(*r*_i_,*r*_j_) even for patterns we have not seen in the past. Indeed, we found that *P*(*s*|*r*) for a held-out test response, *r*, could be well estimated simply by taking *P*(*s*|*r*′) for the response, *r*′, which is most similar to it. Similarity was assessed using the thesaurus that we learned from the training data, that is, for different stimuli than the ones we tested on (Figure 7A,B). This approach clearly improved our ability to estimate the stimulus compared to our prior knowledge about the stimulus, as measured by the Jensen-Shannon divergence between the ‘true’ *P*(*s*|*r*) and the estimate (Figure 7C). Using a thesaurus based on the Hamming distance clearly degraded performance and reduced the accuracy of the stimulus estimate (Figure 7C).

**Figure 7—figure supplement 1.**
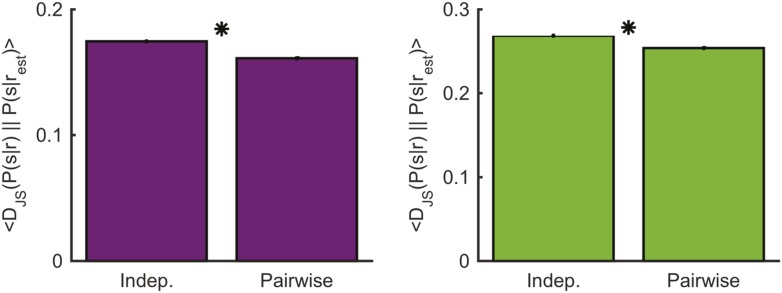
Comparing decoding performance using the conditionally independent model and pairwise model. Left: The average Jensen-Shannon divergence between the ‘true’ P(s|r) and the estimate based on the most similar response as measured using either the conditionally independent model or the pairwise model used in the main text (see Figure 7), for responses evoked by an artificial video. Plotted are mean and standard errors (barely discernable) across all patterns that had at least one close neighbor (<0.25 bits away). The pairwise model performs on average slightly yet significantly better (p < 10^−4^, two-sided paired sign test). Right: Same as left, but for the natural video. Again, the pairwise model performs on average slightly yet significantly better (p < 10^−4^, two-sided paired sign test). **DOI:** http://dx.doi.org/10.7554/eLife.06134.021

Thus, using a very limited set of known responses it is possible to decode novel responses to novel stimuli. This may be crucial when encountering new responses to known stimuli, due to sensory noise, or when generalizing prior knowledge to novel stimuli.

## Discussion

We presented a thesaurus for a neural population code—a similarity measure between population activity patterns, based on the meaning of the responses and not on their syntactic structure. This is a general framework for mapping the codebook in any sensory system, since it makes no assumptions about what makes two spike trains similar, or that the similarity structure should obey a metric in the strict mathematical sense. Indeed, it revealed that the neural code of the brain is very different than our usual mathematical intuition: what we may regard as intuitive measures, like the Hamming distance, fail to capture the similarity between neural population responses. Instead, we found a highly structured codebook, organized in groups of responses that carry very similar information, and are distinct from all other responses.

This organization of the neural population codebook into a relatively small number of semantically different clusters is reminiscent of the design of engineered codes used in communication. There, to overcome noise, different codewords are placed far apart in space of codewords, to allow for error-correction. Since noise may corrupt a message that is sent through a channel, when a pattern is received, it is compared to all possible codewords and is classified according to the nearest one. Importantly, the distance between codewords must be defined by the nature of the noise. Here, we found a neural population code that seems to be built along similar lines. Our thesaurus revealed that most of the information that the population encodes is held by the identity of the cluster that a specific activity pattern belongs to, and that more detailed information may be carried by the fine structure of the pattern. These results generalized across novel stimuli and scaled slowly with the number of stimuli used (Figure 5—figure supplement 1). We thus suggest that our analysis reveals a design principle of the population codebook of a sensory circuit.

What are the advantages of such a population code design? We note that for a large neural population, most network activity patterns are observed at most once (Ganmor et al., 2011a). Thus, the brain frequently receives from its sensory neurons activity patterns that were never seen before. This may occur either due to sensory noise, corrupting the response to a known stimulus but also as a result of a truly novel stimulus. In either case, a semantic similarity measure between neural population responses may explain how these novel inputs may be deciphered: when a novel response pattern is encountered it can take on the information content of similar responses, whose meaning is already known. Therefore, the thesaurus may not only explain how sensory noise can be overcome but also how the brain can generalize across stimuli. Indeed, the thesaurus enabled us to decode neural activity patterns we have not seen before, based on their similarity to other responses.

Uncovering the structure and organization of the population code relied on our ability to accurately model the noisy nature of the population response to specific stimuli. It is the nature of the noise that determines how likely two responses are to be interchanged, and consequently the semantic similarity between them. Here, we used pairwise maximum entropy models to capture the ‘noise correlations’ at every time point, (Figure 2), which were weak on average, but significant at specific times, as was also shown in (Kohn and Smith, 2005; Pillow et al., 2008; Cafaro and Rieke, 2010; Granot-Atedgi et al., 2013).

Interestingly, learning a thesaurus using the conditionally independent model of the neural responses to the stimuli, recovered a similarity structure that was on par with that revealed by the pairwise model, in terms of the information that the clusters carried about the stimulus. These results are surprisingly given that the cells were clearly not conditionally independent. One possible explanation is that the organization of the codebook may be so robust, namely that the separation between clusters is sufficiently large, that even an inaccurate model can capture the identity clusters because the errors of the model are smaller than the distance between clusters. The results of (Schwartz et al., 2012; Granot-Atedgi et al., 2013) suggest that the contribution of noise correlations to shaping the population response to stimuli will increase significantly with network size. Since by definition, the conditional independent model implies larger encoding noise (noise entropy), this suggests that the clusters would be ‘wider’ in this case. It would therefore be most interesting to explore the thesaurus that these models would give for larger populations of cells, beyond groups of 20 cells that we used here for sampling reasons. Finally, we note again that the pairwise models gave better results for decoding stimuli over the conditionally independent model.

Previous studies have suggested mathematically elegant and computationally efficient measures of spike train similarity, relying mostly on edit-distance based metrics (Victor and Purpura, 1997; van Rossum, 2001; Victor, 2005; Houghton and Sen, 2008). Our approach is fundamentally different, as it does not assume a metric structure, or particular features of the code (Haslinger et al., 2013) and does not require assumptions about the syntactic similarity between activity patterns. But maybe most importantly, we found that the semantic similarity between population responses could not be approximated by simple linear or bilinear functions of the patterns. This is mostly due to the fact that noise showed a high degree of stimulus dependence (Figure 3—figure supplement 2). This means that the correlated nature of neural noise shapes the population code differently for different stimuli.

The approach we presented here could be immediately applied to other neural circuits (Bathellier et al., 2012; Parnas et al., 2013), and it is therefore important to note where our approach has been restrictive and could be extended. First, our clustering was not optimized to maximize the information that the clusters carry about the stimulus, but only to group similar responses together. Interestingly, such an information maximization clustering approach (Slonim and Tishby, 1999) does not result in significantly more information using fewer clusters (Figure 5—figure supplement 2). Second, hard clustering of the population responses into *k* clusters is somewhat arbitrary, and it is possible to consider generalizations to fuzzy rather than hard clustering—where responses may be associated with several clusters, or categories, simultaneously in a probabilistic manner (Zemel et al., 1998). Lastly, while both agglomerative and spectral clustering yielded similar results in our analysis of the nature of the retinal population code, it is possible that other ways of clustering would reveal further structure of the code.

## Materials and methods

### Electrophysiology

Experiments were performed on the adult tiger salamander (*Ambystoma tigrinum*). All experiments were approved by the institutional animal care and use committee of Ben-Gurion University of the Negev and were in accordance with government regulations. Prior to the experiment the salamander was adapted to bright light for 30 min. Retinas were isolated from the eye and peeled from the sclera together with the pigment epithelium. Retinas were placed with the ganglion cell layer facing a multi-electrode array with 252 electrodes (Ayanda Biosystems, Lausanne, Switzerland) and superfused with oxygenated (95% O_2_/5% CO_2_) Ringer medium which contains: 110 mM NaCl, 22 mM NaHCO_3_, 2.5 mM KCl, 1 mM CaCl_2_, 1.6 mM MgCl_2_, and 18 mM Glucose, at room temperature (Meister et al., 1994). The electrode diameter was 10 μm and electrode spacing varied between 40 and 80 μm. The array was lowered onto the retina from above by means of a standard mechanical manipulator. Extracellularly recorded signals were amplified (Multi Channel Systems, Germany) and digitized at 10k Samples/s on four personal computers and stored for off-line spike sorting and analysis. Spike sorting was done by extracting the amplitude and width from each potential waveform, and then by clustering using an in-house written MATLAB program (Source code 1). 48/31 retinal ganglion cells were recorded and cleanly isolated in the artificial/natural video experiment.

### Visual Stimulation

Natural video clips were acquired using a digital video camera (Sony Handycam DCR-HC23) at 30 frames per second. The stimulus was projected onto the salamander retina from a CRT video monitor (ViewSonic G90fB) at a frame rate of 60 Hz such that each acquired frame was presented twice, using standard optics (Puchalla et al., 2005). The original color videos were converted to gray scale, using a gamma correction for the computer monitor. Artificial full-field flicker stimuli were generated by sampling a uniform gray level from a normal distribution. In both cases, the visual stimulus covered the retinal patch that was used for the experiment entirely. A 10 s clip was taken from each video and played to separate retinas repetitively for approximately 2 hr. Each video was therefore replayed to the retina over 600 times.

### Data Analysis

Analysis was carried out in MATLAB. We examined the responses of ten randomly chosen groups of 20 neurons from each retina. Spikes were binned at 20 ms (different bin sizes did not qualitatively affect our results, see Figure 5—figure supplement 4). Each response *r* is a 20 bit binary vector with each bit corresponding to the activity of a single neuron (0 representing silence and 1 representing spiking) in a single time bin.

### Estimating the noise distribution

To estimate the probability distribution of responses to a given stimulus *P*(*r*|*s*), we considered the ensemble of responses recorded at a single point in time in the video across repeats. Note that the retina is displayed with the exact same stimulus, including several minutes of stimulus history, at each repeat. We then estimated the single neuron and pairwise spiking probability from the data in order to generate a maximum entropy pairwise model as detailed in previous work (Ganmor et al., 2011b). Briefly, the maximum entropy pairwise distribution is known to take the form (Jaynes, 1957) $P^{\left(2\right)}\left(r|s\right)=\frac{1}{Z\left(s\right)}\text{exp}\left(\overset{N}{\underset{i=1}{\sum }}\alpha_{i}\left(s\right)r_{i}+\underset{i<j}{\sum }\beta_{ij}\left(s\right)r_{i}r_{j}\right)$. The parameters can be found by optimizing the likelihood of the data *L*(Data|*α*,*β*). Since the log likelihood is concave, the global optimum can be found using gradient methods, with local derivatives $\frac{\partial \text{L}}{\partial \alpha_{i}\left(s\right)}={\langle r_{i}\rangle }_{P_{Data}\left(r|s\right)}-{\langle r_{i}\rangle }_{P^{\left(2\right)}\left(r|s\right)}$, where <*f*(*r*)>_*P*_ represents the expected value of some function of the response, *f*, with respect to the probability distribution *P*.

### Clustering and Information

Data were split into train and test sets. The train set was used to learn the conditional probabilities *P*(*r*|*s*), which were then used to construct *P*(*s*|*r*) through Bayes' rule *P*(*s*|*r*) = *P*(*r*|*s*)*P*(*s*)/*P*(*r*). The dissimilarity matrix between all responses observed in the test set was defined as *d*(*r*_1_,*r*_2_) = *D*_JS_(*P*(*s*|*r*_1_)||*P*(*s*|*r*_2_)) (where *D*_JS_ stands for the Jensen-Shannon divergence, and *s* represents only train stimuli). This matrix was clustered using hierarchical agglomerative clustering with the distance between clusters defined as the average distance between inter-cluster pairs. Different methods of spectral clustering yielded similar results. The number of clusters, *k*, was systematically varied. For a given value of *k*, the mutual information between stimulus and clusters *I*(*s*;*C*_*k*_(*r*)) was estimated as follows: Each response in the test data was replaced with its cluster label *C*_*k*_(*r*) (*C*_*k*_(*r*) can take a value in {1…*k*}) and then mutual information was estimated as *I*(*s*;*C*_*k*_(*r*)) = *H*(*C*_*k*_(*r*)) − *H*(*C*_*k*_(*r*)|*s*), where *H* is the Shannon entropy. *H*(*C*_*k*_(*r*)) is the total entropy of different clusters in the data. $H\left(C_{k}\left(r\right)\right)=-\overset{k}{\underset{i=1}{\sum }}P\left(C_{k}\left(r\right)=i\right)\log\left(P\left(C_{k}\left(r\right)=i\right)\right)$, where $P\left(C_{k}\left(r\right)=i\right)$ is the frequency of the *i*th cluster, out of *k*, in the data. *H*(*C*_*k*_(*r*)|*s*) is the average conditional entropy of clusters given stimulus *s*. In our case, we treat each time point in the 10 s video as a unique stimulus, and thus *H*(*C*_*k*_(*r*)|*s*) is given by the conditional entropy at each time point across video repeats, averaged over time points—$H\left(C_{k}\left(r\right)\text{|}s\right)=H\left(C_{k}\left(r\right)\text{|}t\right)=-\overset{T}{\underset{t=1}{\sum }}\frac{1}{T}\overset{k}{\underset{i=1}{\sum }}P\left(C_{k}\left(r\right)=i\text{|}t\right)\log P\left(C_{k}\left(r\right)=i\text{|}t\right)$, where $P\left(C_{k}\left(r\right)=i|t\right)$ is the frequency of cluster *i*, out of *k*, at time *t* in the video. The representative matrices in Figure 3 were chosen as the matrices with the fifth (out of 10) highest average ratio of the within cluster similarity and the overall similarity. The total degree of network correlation was measured using the multi-information (Amari, 2001; Schneidman et al., 2003b), defined as *I*_N_ = *H*_ind_ − *H*, where *H*_ind_ is the independent entropy (sum of all individual neuron entropies) and *H* is the entropy estimated from the actual data. In all cases where information or entropy was estimated, we used the method of extrapolation (Treves and Panzeri, 1995; Strong et al., 1998) to correct for finite sampling biases. These corrections were on the order of a few percent (see Figure 2—figure supplement 1).

### Decoding

For a given response *r* in the test set, we compared the ‘true’ *P*(*s*|*r*), constructed from *P*(*r*|*s*) across test stimuli as previously described, to one of three estimates $\tilde{P}\left(s\text{|}r\right)$: (1) The true prior over stimuli *P*(*s*), which is a uniform distribution over stimuli. (2) A nearest neighbor estimate, that is, $\tilde{P}\left(s\text{|}r\right)$ = *P*(*s*|*r*′) where *d*(*r*,*r*′) is minimal across all responses in the test set. (3) Same as 2, only r′ is a nearest neighbor in Hamming distance (arbitrarily chosen out of all nearest neighbors).

### Response embedding

Responses were embedded using the *Isomap* algorithm (Tenenbaum et al., 2000). Given a set of pairwise distances, *Isomap* finds a configuration of points in *k* dimensions that most closely recreate the geodesic distances between points. The geodesic distance between two points is either the dissimilarity *d* between them, if they are connected in the neighborhood graph, or the shortest weighted path if they are not. Two nodes were connected if their dissimilarity was smaller than 0.5 bits (epsilon neighborhood). *Isomap* was chosen over multi dimensional scaling approaches as it gave better results, in particular since dissimilarities are bounded from above (1 bit maximum). We limited the number of points to embed so that a 2–3 dimensional embedding gave a reasonably faithful reconstruction of the geodesic distances as measured by the residual variance.

## Additional files

Source code 1. MATLAB code used in the clustering and information analyses. This .m file contains functions used in the analyses presented in the paper. It is not intended to be run as is, instead each function needs to be copied and placed in its own .m file. These functions are also available at the following GitHub respository - https://github.com/eladganmor/Neural_Thesaurus **DOI:** http://dx.doi.org/10.7554/eLife.06134.022

## References

[bib1] Abbott LF, Dayan P (1999). The effect of correlated variability on the accuracy of a population code. Neural Computation.

[bib2] Ala-Laurila P, Greschner M, Chichilnisky EJ, Rieke F (2011). Cone photoreceptor contributions to noise and correlations in the retinal output. Nature Neuroscience.

[bib3] Amari S (2001). Information geometry on hierarchy of probability distributions. IEEE Transactions on Information Theory.

[bib4] Amari S, Nakahara H, Wu S, Sakai Y (2003). Synchronous firing and higher-order interactions in neuron pool. Neural Computation.

[bib5] Averbeck BB, Latham PE, Pouget A (2006). Neural correlations, population coding and computation. Nature Reviews Neuroscience.

[bib6] Bathellier B, Ushakova L, Rumpel S (2012). Discrete neocortical dynamics predict behavioral categorization of sounds. Neuron.

[bib7] Berry MJ, Warland DK, Meister M (1997). The structure and precision of retinal spike trains. Proceedings of the National Academy of Sciences of USA.

[bib8] Bohte SM, Spekreijse H, Roelfsema PR (2000). The effects of pair-wise and higher order correlations on the firing rate of a post-synaptic neuron. Neural Computation.

[bib9] Borst A, Theunissen FE (1999). Information theory and neural coding. Nature Neuroscience.

[bib10] Cafaro J, Rieke F (2010). Noise correlations improve response fidelity and stimulus encoding. Nature.

[bib11] Cover TM, Thomas JA (1991). Elements of information theory.

[bib12] Curto C, Itskov V, Morrison K, Roth Z, Walker JL (2013). Combinatorial neural codes from a mathematical coding theory perspective. Neural Computation.

[bib13] Dan Y, Alonso JM, Usrey WM, Reid RC (1998). Coding of visual information by precisely correlated spikes in the lateral geniculate nucleus. Nature Neuroscience.

[bib14] de la Rocha J, Doiron B, Shea-Brown E, Josić K, Reyes A (2007). Correlation between neural spike trains increases with firing rate. Nature.

[bib15] de Ruyter van Steveninck RR, Lewen GD, Strong SP, Koberle R, Bialek W (1997). Reproducibility and variability in neural spike trains. Science.

[bib16] Diesmann M, Gewaltig MO, Aertsen A (1999). Stable propagation of synchronous spiking in cortical neural networks. Nature.

[bib17] Ecker AS, Berens P, Keliris GA, Bethge M, Logothetis NK, Tolias AS (2010). Decorrelated neuronal firing in cortical microcircuits. Science.

[bib18] Faisal AA, Selen LP, Wolpert DM (2008). Noise in the nervous system. Nature Reviews Neuroscience.

[bib19] Fujisawa S, Amarasingham A, Harrison MT, Buzsaki G (2008). Behavior-dependent short-term assembly dynamics in the medial prefrontal cortex. Nature Neuroscience.

[bib20] Ganmor E, Segev R, Schneidman E (2011a). Sparse low-order interaction network underlies a highly correlated and learnable neural population code. Proceedings of the National Academy of Sciences of USA.

[bib21] Ganmor E, Segev R, Schneidman E (2011b). The architecture of functional interaction networks in the retina. The Journal of Neuroscience.

[bib22] Granot-Atedgi E, Tkačik G, Segev R, Schneidman E (2013). Stimulus-dependent maximum entropy models of neural population codes. PLOS Computational Biology.

[bib23] Harvey CD, Coen P, Tank DW (2012). Choice-specific sequences in parietal cortex during a virtual-navigation decision task. Nature.

[bib24] Haslinger R, Pipa G, Lewis LD, Nikolić D, Williams Z, Brown E (2013). Encoding through patterns: Regression tree–based neuronal population models. Neural Computation.

[bib25] Hopfield JJ (1982). Neural networks and physical systems with emergent collective computational abilities. Proceedings of the National Academy of Sciences of USA.

[bib26] Houghton C, Sen K (2008). A new multineuron spike train metric. Neural Computation.

[bib27] Hubert L, Schultz J (2011). Quadratic assignment as a general data analysis strategy. The British Journal of Mathematical and Statistical Psychology.

[bib28] Huth AG, Nishimoto S, Vu AT, Gallant JL (2012). A continuous semantic space describes the representation of thousands of object and action categories across the human brain. Neuron.

[bib29] Jaynes ET (1957). Information theory and statistical Mechanics. Physical Review.

[bib30] Kohn A, Smith MA (2005). Stimulus dependence of neuronal correlation in primary visual cortex of the Macaque. The Journal of Neuroscience.

[bib31] Loebel A, Nelken I, Tsodyks M (2007). Processing of sounds by population spikes in a model of primary auditory cortex. Frontier in Neuroscience.

[bib32] Mainen ZF, Sejnowski TJ (1995). Reliability of spike timing in neocortical neurons. Science.

[bib33] Maynard EM, Hatsopoulos NG, Ojakangas CL, Acuna BD, Sanes JN, Normann RA, Donoghue JP (1999). Neuronal interactions improve cortical population coding of movement direction. The Journal of Neuroscience.

[bib34] Mazor O, Laurent G (2005). Transient dynamics versus fixed points in odor representations by Locust antennal lobe projection neurons. Neuron.

[bib35] Meister M, Pine J, Baylor DA (1994). Multi-neuronal signals from the retina: acquisition and analysis. Journal of Neuroscience Methods.

[bib36] Nicolelis MA, Baccala LA, Lin RC, Chapin JK (1995). Sensorimotor encoding by synchronous neural ensemble activity at multiple levels of the somatosensory system. Science.

[bib37] Nirenberg S, Carcieri SM, Jacobs AL, Latham PE (2001). Retinal ganglion cells act largely as independent encoders. Nature.

[bib38] Ohiorhenuan IE, Mechler F, Purpura KP, Schmid AM, Hu Q, Victor JD (2010). Sparse coding and high-order correlations in fine-scale cortical networks. Nature.

[bib39] Oizumi M, Ishii T, Ishibashi K, Hosoya T, Okada M (2010). Mismatched decoding in the brain. The Journal of Neuroscience.

[bib40] Osborne LC, Lisberger SG, Bialek W (2005). A sensory source for motor variation. Nature.

[bib41] Parnas M, Lin AC, Huetteroth W, Miesenböck G (2013). Odor discrimination in *Drosophila*: from neural population codes to behavior. Neuron.

[bib42] Pereira F, Tishby N, Lee L (1993). Distributional clustering of english words, in: proceedings of the 31st annual meeting on association for computational linguistics, ACL '93.

[bib43] Pillow JW, Shlens J, Paninski L, Sher A, Litke AM, Chichilnisky EJ, Simoncelli EP (2008). Spatio-temporal correlations and visual signalling in a complete neuronal population. Nature.

[bib44] Pola G, Thiele A, Hoffmann KP, Panzeri S (2003). An exact method to quantify the information transmitted by different mechanisms of correlational coding. Network.

[bib45] Puchalla JL, Schneidman E, Harris RA, Berry MJ (2005). Redundancy in the population code of the retina. Neuron.

[bib46] Reich DS, Mechler F, Victor JD (2001). Independent and redundant information in nearby cortical neurons. Science.

[bib47] Rieke F, Warland D, de Ruyter van Steveninck R, Bialek W (1999). Spikes: exploring the neural code.

[bib48] Rolls ET, Treves A (2011). The neuronal encoding of information in the brain. Progress in Neurobiology.

[bib49] Schneidman E, Berry MJ, Segev R, Bialek W (2006). Weak pairwise correlations imply strongly correlated network states in a neural population. Nature.

[bib50] Schneidman E, Bialek W, Berry MJ (2003a). Synergy, redundancy, and independence in population codes. The Journal of Neuroscience.

[bib51] Schneidman E, Freedman B, Segev I (1998). Ion channel stochasticity may be critical in determining the reliability and precision of spike timing. Neural Computation.

[bib52] Schneidman E, Still S, Berry MJ, Bialek W (2003b). Network information and connected correlations. Physical Review Letters.

[bib53] Schwartz G, Macke J, Amodei D, Tang H, Berry MJ (2012). Low error discrimination using a correlated population code. Journal of Neurophysiology.

[bib54] Shlens J, Field GD, Gauthier JL, Greschner M, Sher A, Litke AM, Chichilnisky EJ (2009). The structure of large-scale synchronized firing in primate retina. The Journal of Neuroscience.

[bib55] Slonim N, Tishby N (1999). Agglomerative information bottleneck.

[bib56] Sompolinsky H, Yoon H, Kang K, Shamir M (2001). Population coding in neuronal systems with correlated noise. Physical Review. E, Statistical, Nonlinear, and Soft Matter Physics.

[bib57] Sreenivasan S, Fiete I (2011). Grid cells generate an analog error-correcting code for singularly precise neural computation. Nature Neuroscience.

[bib58] Stocker AA, Simoncelli EP (2006). Noise characteristics and prior expectations in human visual speed perception. Nature Neuroscience.

[bib59] Strong SP, Koberle R, de Ruyter van Steveninck RR, Bialek W (1998). Entropy and information in neural spike trains. Physical Review Letters.

[bib60] Tenenbaum JB, de Silva V, Langford JC (2000). A global geometric framework for nonlinear dimensionality reduction. Science.

[bib61] Tkačik G, Granot-Atedgi E, Segev R, Schneidman E (2013). Retinal metric: a stimulus distance measure derived from population neural responses. Physical Review Letters.

[bib62] Tkacik G, Schneidman E, Berry MJ, Bialek W (2006). Ising models for networks of real neurons.

[bib63] Treves A, Panzeri S (1995). The upward bias in measures of information derived from limited data samples. Neural Computation.

[bib64] Truccolo W, Hochberg LR, Donoghue JP (2010). Collective dynamics in human and monkey sensorimotor cortex: predicting single neuron spikes. Nature Neuroscience.

[bib65] van Rossum MC (2001). A novel spike distance. Neural Computation.

[bib66] Vargas-Irwin CE, Shakhnarovich G, Yadollahpour P, Mislow JM, Black MJ, Donoghue JP (2010). Decoding complete reach and grasp actions from local primary motor cortex populations. The Journal of Neuroscience.

[bib67] Victor JD (2005). Spike train metrics. Current Opinion in Neurobiology.

[bib68] Victor JD, Purpura KP (1997). Metric-space analysis of spike trains: theory, algorithms and application. Network Computation in Neural Systems.

[bib69] Vidne M, Ahmadian Y, Shlens J, Pillow JW, Kulkarni J, Litke AM, Chichilnisky EJ, Simoncelli E, Paninski L (2012). Modeling the impact of common noise inputs on the network activity of retinal ganglion cells. Journal of Computational Neuroscience.

[bib70] Warland DK, Reinagel P, Meister M (1997). Decoding visual information from a population of retinal ganglion cells. Journal of Neurophysiology.

[bib71] Zemel RS, Dayan P, Pouget A (1998). Probabilistic interpretation of population codes. Neural Computation.

[bib72] Zohary E, Shadlen MN, Newsome WT (1994). Correlated neuronal discharge rate and its implications for psychophysical performance. Nature.

